# Sodium Valproate Versus Levetiracetam in Pediatric Generalized Epilepsy: A Comparative Study

**DOI:** 10.7759/cureus.100114

**Published:** 2025-12-26

**Authors:** Ishan Mahajan, Prabhat Kumar, Ankit Jain, Utkarsh Bansal, Vijay Singh, Ekansh Rathoria

**Affiliations:** 1 Pediatrics, Hind Institute of Medical Sciences, Barabanki, IND; 2 Pediatrics, Hind Institute of Medical Sciences Ataria Sitapur, Sitapur, IND

**Keywords:** aed (anti-epileptic drugs), antiseizure medication, childhood epilepsy, generalized epilepsy, generalized seizure, generalized tonic-clonic seizures, newer antiepileptic drugs, pediatric epilepsy, seizure-free duration, seizure-free outcome

## Abstract

Aim and objectives

Generalized epilepsy impacts a significant portion of the pediatric population. Safety and efficacy are key considerations in the treatment of epileptic seizures in children. New generation anti-seizure medications (ASMs) promise to fulfil this need. Thus, this study was planned to compare the efficacy, safety, and tolerability of sodium valproate (VPA) and levetiracetam (LEV) in children with generalized epilepsy.

Material and methods

A total of 211 pediatric patients aged 2-14 years with generalized seizures were prospectively enrolled and randomized either to receive VPA (n=107) or LEV (n=104) as per the recommended protocol up to six months. Seizure-free duration and seizure-free outcomes were assessed at six months. Adverse events, drug tolerability, and compliance were recorded. The data was statistically analyzed.

Results

Of the 211 participants, 187 completed the study (VPA group: 94; LEV group: 93). The two groups were comparable for demographic and clinical profiles at the time of start of treatment. The seizure-free duration was significantly longer in the VPA group (153.61 ± 53.12 days) compared to the LEV group (135.82 ± 68.79 days). Seizure-free outcome rates at three and six months were 88.3% and 79.8% in the VPA group, and 81.7% and 66.7% in the LEV group, respectively, with a statistically significant difference between the groups at six months. The VPA group had significantly shorter mean hospital need (1.68 days) as compared to the LEV group (2.82 days). Post-treatment increase in SGPT was significantly higher in the VPA group. Nausea/vomiting and weight gain were significantly more common in the VPA group, while behavioral adverse effects were significantly more common in the LEV group.

Conclusion

VPA was found to be more efficacious in controlling seizures in children with generalized epilepsy than LEV. With respect to safety, both treatments had specific issues.

## Introduction

Epilepsy is a chronic neurological disorder affecting over 50 million people worldwide, of which nearly one-third are children [1]. Childhood epilepsy not only imposes a significant medical burden but also affects the psychosocial and cognitive development of affected children, contributing to reduced quality of life and school performance [2]. Among various types of epilepsy, generalized epilepsy, characterized by bilateral synchronous seizure activity involving both cortical and subcortical structures, forms a critical subset in pediatric neurology [3].

Generalized seizures include tonic-clonic, absence, and other (myoclonic, clonic, epileptic spasms, tonic, and atonic) types and often require broad-spectrum antiseizure medications (ASMs), capable of addressing multiple seizure types simultaneously [4].

Sodium valproate (VPA) has long been regarded as a cornerstone in the treatment of generalized epilepsies due to its broad efficacy spectrum. Its mechanisms of action include increased gamma-aminobutyric acid (GABA) availability through inhibition of GABA transaminase, as well as modulation of voltage-gated sodium and calcium channels [5]. However, VPA is associated with significant adverse effects, including hepatotoxicity, weight gain, acute pancreatitis, and aplastic anemia [6]. In the female population, VPA poses a substantial teratogenic risk, with known associations with neural tube defects and impaired cognitive outcomes in offspring when used during pregnancy [7]. Due to these safety concerns, there has been increasing interest in alternative ASMs with more favorable side effect profiles.

One such alternative is levetiracetam (LEV), a relatively newer antiepileptic agent that has gained popularity for its broad-spectrum efficacy, minimal hepatic metabolism, and favorable pharmacokinetics [8]. LEV exerts its antiepileptic action primarily through binding to the synaptic vesicle protein SV2A, modulating neurotransmitter release and neuronal excitability [9]. It exhibits linear kinetics, negligible protein binding, and minimal drug-drug interactions, making it particularly suitable for pediatric use where polytherapy is often required. Importantly, LEV has a reduced need for laboratory monitoring compared to VPA and is considered more tolerable in terms of systemic toxicity.

Few clinical studies have attempted to compare LEV and VPA directly in pediatric populations. For instance, Latif et al. conducted a randomized controlled trial in children aged 3 to 12 years with epilepsy (both focal and generalized types) and found that seizure control was achieved in 85% of the LEV group versus 73% in the VPA group over six months of follow-up [10]. Additionally, fewer patients in the LEV group experienced side effects (31%) compared to the VPA group (90%), with significantly less weight gain and hepatotoxicity observed [10]. Similarly, Bhayana et al. conducted a randomized open-label trial in children with epilepsy and reported comparable efficacy between LEV and VPA, though behavioral side effects such as irritability and mood changes were more frequent in the LEV group [11].

Conversely, results from larger pragmatic trials such as the SANAD II study- a multicenter randomized controlled trial-suggested that VPA was more effective than LEV in achieving 12-month seizure remission in generalized epilepsy patients, although most participants were adolescents or adults [12]. While SANAD II is highly influential, its generalizability to younger pediatric populations, particularly those under 12 years, remains limited due to differences in seizure types, developmental concerns, and medication metabolism.

Despite increasing use of LEV, comparative studies specifically in children with generalized epilepsy remain sparse, and few trials have stratified results by epilepsy syndrome (e.g., juvenile myoclonic epilepsy vs. absence epilepsy) or included long-term cognitive, behavioral, and quality-of-life outcomes. A recent study by Fattahzadeh et al. retrospectively compared LEV and VPA in Iranian children with generalized epilepsy and found better seizure control in the VPA group, but higher tolerability and fewer side effects in the LEV group [13].

Given the chronic nature of epilepsy and the critical developmental window in childhood, the choice of antiepileptic therapy must be carefully balanced between seizure control and safety/tolerability. Major comparative trials (e.g., SANAD II) predominantly included adults and adolescents, significantly limiting their generalizability to younger pediatric populations. Our study aims to specifically target children aged 2-14 years, a critical group with distinct metabolic and developmental considerations, to compare the efficacy of VPA and LEV in the treatment of generalized epilepsy. Furthermore, we aim for a direct, quantified comparison of both pharmacoeconomic metrics and specific side effect profiles in a developing country setting, which is underrepresented in the current literature.

## Materials and methods

Study design and setting

This was an institution-based, comparative analytical study conducted in the Department of Pediatrics at the Hind Institute of Medical Sciences, Safedabad, Barabanki, a tertiary care center in Uttar Pradesh, India. The duration of the study was 12 months, following approval from the Institutional Ethical Committee. The research aimed to evaluate and compare the efficacy, safety, and tolerability of LEV and VPA in pediatric patients with newly diagnosed or inadequately treated generalized epilepsy.

Study population

The study included children aged 2-14 years who were diagnosed with generalized epilepsy, defined as patient has had at least two unprovoked (or reflex) seizures occurring more than 24 hours apart; has had a single unprovoked (or reflex) seizure but faces a high likelihood of further seizures, with a recurrence risk comparable to that seen after two unprovoked seizures (at least 60%) over the next 10 years; or has a diagnosed epilepsy syndrome [14]. Eligible participants were either newly diagnosed or had been inadequately treated before enrollment. Children were excluded from the study if they had a known hypersensitivity to the study medications, a history of chronic liver disease, metabolic disorders, or structural brain abnormalities. Additionally, participants who had previously used any of the study drugs were not eligible for inclusion. Inadequately treated generalized epilepsy was defined as an incorrect diagnosis or inappropriate medication selection in a child already on epilepsy treatment.

Sample size and randomization

The sample size was determined based on previously reported differences in the mean time to first seizure between VPA and LEV monotherapy in pediatric epilepsy. According to Bhayana et al., the mean (± SD) time to first seizure was 53.75 ± 54.64 days in the VPA group and 34.36 ± 36.45 days in the LEV group [11]. Assuming a two-tailed significance level (α) of 0.05, power (1−β) of 80%, and an equal allocation ratio between groups, the sample size was calculated using the formula for comparing two independent means:

\(n = \frac{\left(Z_{1-\alpha/2} + Z_{1-\beta}\right)^2 \left(\sigma_1^2 + \sigma_2^2\right)}{(\mu_1 - \mu_2)^2} \)

Where μ_1_ and μ_2_ are the group means, σ_1_ and σ_2_ are the corresponding standard deviations, Z_(1-α/2) _= 1.96 and Z_(1-β) _= 0.84 for the stated confidence and power levels.

Substituting the values:

$n = \frac{(1.96 + 0.84)^2 \times (54.64^2 + 36.45^2)}{(53.75 - 34.36)^2}$ ≈ 90 participants per group

Thus, the minimum required sample size was 180 children (90 per group). To account for potential attrition, dropouts, or protocol violations, a total of 211 children were enrolled and randomized in a 1:1 allocation ratio into two intervention arms: the VPA group and the LEV group. 

Randomization was carried out using a computer-generated random number sequence, employing simple randomization without stratification or blocking, using Microsoft Excel (Microsoft Corporation, Redmond, WA, United States) by applying the appropriate algorithm. Allocation was concealed until the point of intervention assignment.

Intervention Protocol

Patients in both groups were managed according to a standard care protocol, comprising a baseline assessment and follow-up visits.

Baseline Assessment

**Figure 1:**
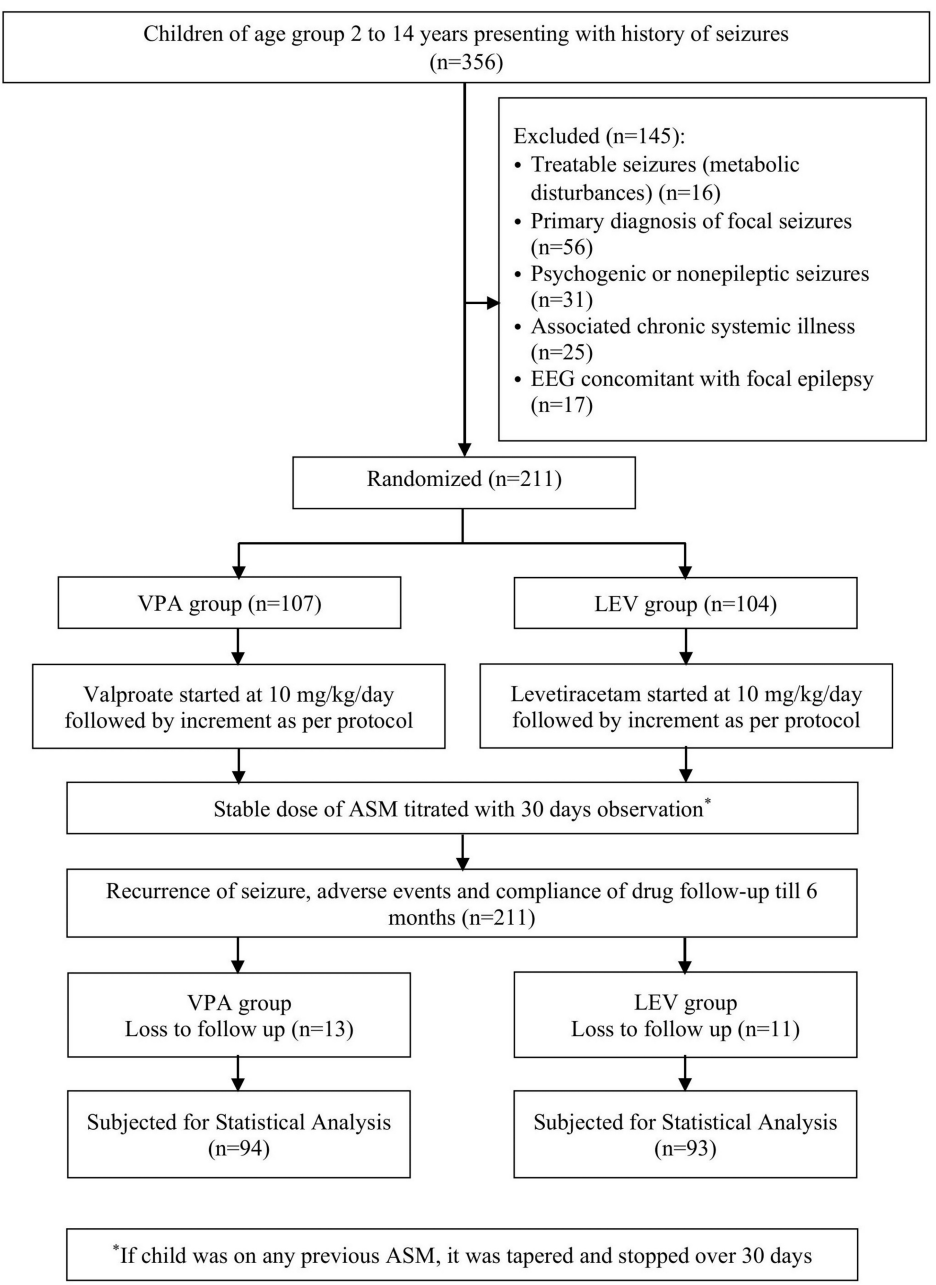
Flow of Recruitment of Participants in the Study EEG: electroencephalogram; VPA: sodium valproate; LEV: levetiracetam; ASM: anti-seizure medication

Detailed clinical evaluation, anthropometry (weight, height, BMI), including a detailed history of the seizure semiology, including the type, number of seizure episodes in the last month, age at onset, details of previous ASM, electroencephalogram (EEG), and laboratory investigations (CBC, liver function tests, serum amylase). The duration of hospitalization to achieve seizure control was noted. Behavioral assessments were conducted at baseline and during follow-up visits using a checklist to monitor potential changes in behavior. The VPA group received a loading dose of 20 mg/kg followed by 10 mg/kg/day, and titrated up by 10 mg/kg/day over 1-2 weeks to a maximum dosage of 60 mg/kg/day (maximum 1000 mg/day), if needed. While in the LEV group, a loading dose of 60 mg/kg was given, followed by 20 mg/kg/day, titrated up by 10 mg/kg/day over 1-2 weeks to a maximum dosage of 60 mg/kg/day (maximum 3000 mg/day), if required, as shown in Figure 1 [15]. No changes or additions of concomitant ASMs were allowed during the study, and all earlier ASMs (if any) were tapered and discontinued over 30 days before the start of the study period.

Follow-up Visits

Scheduled monthly for six months. EEG and laboratory tests were repeated at three- and six-month intervals. Seizure control, adverse events, and drug tolerability were monitored continuously. Adverse events were monitored using a structured checklist administered by the investigator during follow-up visits. This assessment specifically covered common side effects, including behavioral changes (aggression, irritability), gastrointestinal issues (nausea, vomiting), and neurological signs (somnolence). Compliance was reinforced and assessed during follow-up.

Outcome measures

The primary outcomes of the study included the duration of seizure-free periods and the proportion of children who remained seizure-free at three and six months. Secondary outcomes assessed adverse events-covering behavioral, gastrointestinal, neurological, and hepatic domains-along with changes in serum glutamic pyruvic transaminase (SGPT) levels at different follow-up intervals. Additional measures involved evaluating the duration of hospital stay during the initial treatment phase and assessing overall drug tolerability and compliance among participants. Seizure-free duration was defined as the time period from discharge to the repeat seizure activity. Seizure freedom was defined as the complete absence of clinical seizure episodes during the follow-up period, as documented through patient/caregiver reports and verified by clinical evaluation.

Ethical considerations

The study protocol was reviewed and approved by the Institutional Ethics Committee (IEC) of Hind Institute of Medical Sciences. Ethical principles were adhered to as per the Declaration of Helsinki. Each participant's parent or legal guardian provided written informed consent. Confidentiality of participant data was strictly maintained using unique patient identification numbers.

Data collection and analysis

Data was recorded using a semi-structured proforma and digitized using Microsoft Excel 2023. Statistical analysis was performed using IBM SPSS version 22.0 (IBM Corp., Armonk, NY). Continuous variables were presented as mean ± standard deviation (SD). Categorical variables were summarized as frequencies and percentages. Inter-group comparisons were made using the chi-square test for categorical data and the Student’s t-test for continuous variables. Paired t-tests were used to assess intra-group changes. A p-value <0.05 was considered statistically significant.

## Results

According to the inclusion and exclusion criteria, 211 participants were screened and enrolled. A total of 24 patients were lost to follow-up. A total of 187 children completed the study, consisting of 118 boys (63.1%) and 69 girls (36.9%), with a mean age of 6.72 ± 2.93 years. The mean age of onset for generalized seizures was 6.61 ± 2.93 years. A significant proportion of patients, 145 (77.5%), were treatment-naive. Among those who had received a prior ASM, carbamazepine was the most commonly used, accounting for 22 (11.8%) of the cases. There was no statistically significant difference between the two study groups (VPA vs LEV) in terms of age, sex distribution, age of onset, anthropometric data, and history of ASM use at the time of enrollment (Table 1).

**Table 1:** Baseline Characteristics of the Enrolled Participants Data are presented as n (%) for categorical variables and as mean ± SDᵃ (age range: 2–14 years) for continuous variables. The chi-square test was used to calculate the p-value for categorical data, and the t-test was used to calculate the p-value for continuous data. VPA: sodium valproate; LEV: levetiracetam; SD: standard deviation

Characteristics	VPA group (n=94)	LEV group (n= 93)	Statistical test value	p-value
Male	56 (59.6)	62 (66.6)	ꭓ2 = 1.010	0.315
Mean age (years)^a^	6.34 ± 2.68	7.12 ± 3.13	t = 1.826	0.069
Mean age at onset (years)^a^	6.20 ± 2.66	7.03 ± 3.13	t = 1.951	0.053
BMI (kg/m^2^)^a^	15.35 ± 1.40	15.42 ± 1.94	t = -1.756	0.081
Previous anti-seizure medication				
None	76 (80.9)	69 (74.2)	ꭓ2 = 1.403	0.405
Carbamazepine	10 (10.6)	12 (12.9)		
Oxcarbazepine	7 (7.4)	11 (11.8)		
Phenobarbital	1 (1.1)	1 (1.1)		

The mean duration of hospitalization was shorter in the Group VPA 1.68 ± 0.94 days, compared to 2.82 ± 0.79 days in the LEV group (t = 10.999, p < 0.001). The median (IQR) time to seizure-free period after hospitalization was 2 (1-2) days in the VPA group and 3 (2-3) days in the LEV group, with significantly faster response in the VPA group.

The seizure-free duration was 153.61 (95% CI: 142.73, 164.49) days in the VPA group compared to 135.82 (95% CI: 121.65, 149.99) days in the LEV group (t = 1.99, p = 0.048). The proportion of seizure-free patients at six months was 79.8% (n = 75) in the VPA group and 66.7% (n = 62) in the LEV group. There was a statistically significant difference between the two groups in terms of seizure-free outcomes, favoring VPA (p = 0.043) as shown in Table 2.

**Table 2:** Proportion of Patients Remaining Seizure-free at 3 and 6 Months Data presented as n (%). VPA: sodium valproate; LEV: levetiracetam

Follow-up interval	VPA group (n=94)	LEV group (n= 93)	Total (n=187)	ꭓ2 value	p-value
3 months	83 (88.3)	76 (81.7)	159 (85.0)	1.580	0.209
6 months	75 (79.8)	62 (66.7)	137 (73.3)	4.086	0.043

Mean dose to control seizure in the VPA group was 17.23±7.78 mg/kg/day, while that required in the LEV group was 23.55±9.17 mg/kg/day. Adverse events were observed in 34/94 (36.2%) patients of the VPA group and 24/93 (25.8%) patients of the LEV group, with no statistically significant difference between the groups (Table 3). In the VPA group, there were more metabolic side effects (nausea and vomiting, as well as weight gain). In the LEV group, there were more behavioral side effects (aggression, irritability). Somnolence was seen in both groups. Importantly, adverse events were mild, and there were no treatment withdrawals in either group, demonstrating 100% drug tolerability for both VPA and LEV. There was no statistically significant correlation seen between dose and adverse events in both groups.

**Table 3:** Comparison of Adverse Events Between the Two Study Groups Data presented as n (%). VPA: sodium valproate; LEV: levetiracetam

Adverse effect	VPA group (n=94)	LEV group (n= 93)	ꭓ2 value	p-value
No adverse effect	60 (63.8%)	69 (74.2%)	2.347	0.126
Somnolence	11 (11.7%)	4 (4.3%)	3.471	0.062
Aggression	0 (0.0%)	9 (9.7%)	9.557	0.002
Irritability	0 (0.0%)	8 (8.6%)	8.447	0.004
Nausea & vomiting	13 (13.8%)	3 (3.2%)	6.719	0.010
Weight gain	10 (10.6%)	0 (0.0%)	10.453	0.001

Both groups showed a rise in SGPT over time, but the increase was significantly higher in the VPA group, indicating more hepatotoxic potential (Table 4).

**Table 4:** Comparison of SGPT Levels Between the Two Study Groups Data presented as mean ± SD. VPA: sodium valproate; LEV: levetiracetam; SGPT: serum glutamic pyruvic transaminase

Time Point	VPA group (n=94)	LEV group (n= 93)	t-value	p-value
Baseline	14.61 ± 3.69	14.52 ± 3.63	0.169	0.866
3 Months	24.13 ± 6.68	18.32 ± 5.12	6.664	<0.001
6 Months	30.70 ± 14.39	20.87 ± 5.87	6.105	<0.001

An analysis of covariance (ANCOVA) was performed to examine differences in BMI at six months between children treated with valproate and levetiracetam, controlling for baseline BMI. The overall model was statistically significant (F(2, 184) = 299.78, p < 0.001, R² = 0.765), with both treatment group and baseline BMI significantly predicting BMI at six months (Table 5).

**Table 5:** Comparison of BMI Between the Two Study Groups Note: Results are adjusted using ANCOVA, controlling for baseline BMI (held at mean = 15.39). VPA: sodium valproate; LEV: levetiracetam; BMI: body mass index

Treatment Group	Adjusted Mean BMI	SE	95% CI	Mean Difference	p (Bonferroni)	Partial η²
VPA	16.75	0.15	16.45, 17.04	+1.19 (vs LEV)	< .001	0.147
LEV	15.56	0.15	15.26, 15.85	—	—	—

Children in the VPA group had a significantly higher adjusted BMI at six months than those in the LEV group (mean difference = 1.19, p < 0.001), indicating a moderate-to-large effect size (partial η² = 0.147), even after adjusting for initial BMI.

## Discussion

The most common onset of pediatric epilepsy observed is generalized in various studies, and specifically in this region found to be 58.4% by Chagantipati et al. and 79.3% by Sharma et al. in their studies [16, 17]. The present study provides a robust comparative evaluation of LEV and VPA as first-line antiepileptic agents in pediatric patients with generalized epilepsy. The findings reveal that while both medications are effective in achieving seizure control, VPA demonstrated statistically superior outcomes in terms of longer seizure-free duration, quicker onset of seizure control, and shorter hospital stays. Nonetheless, LEV emerged as a clinically valuable alternative, especially in children with concerns about VPA's safety or tolerability. The superior efficacy of VPA can be attributed to its broad-spectrum antiepileptic properties and its ability to enhance GABAergic neurotransmission, thereby stabilizing both cortical and subcortical neuronal circuits implicated in generalized epilepsies. 

In terms of efficacy, the mean seizure-free duration in the VPA group was significantly longer at 153.6 days, compared to 135.8 days in the LEV group, with a p-value of 0.049, indicating statistical significance. This is consistent with the findings from Latif et al., who, in a randomized controlled trial, reported that 76% of children treated with VPA achieved complete seizure freedom within 6 months, compared to only 62% in the LEV group [9]. Similarly, Meena et al., in a large-scale RCT conducted in India, found higher seizure-free rates with VPA (81.3%) compared to LEV (72.5%) over a six-month follow-up period [18]. Our study offers new, critical insights by focusing exclusively on the pediatric population aged 2-14 years, providing age-specific data often diluted or absent in major adult/adolescent trials.

Another key finding was that the mean hospital stay was significantly shorter in the VPA group (1.68 days) compared to the LEV group (2.82 days), indicating a more rapid onset of clinical efficacy with VPA. These results corroborate the findings of Vignesh et al., who reported a mean hospital stay of 5.5 days for pediatric status epilepticus patients treated with VPA versus 7 days for those treated with LEV [19]. Such pharmacoeconomic considerations are particularly critical in developing countries like India, where the affordability of long-term treatment poses a significant challenge.

However, differences in safety and tolerability were evident. VPA was associated with a higher incidence of gastrointestinal and metabolic side effects, including nausea, vomiting, and weight gain, as well as a significant increase in SGPT levels at 3 and 6 months (p < 0.001), suggesting potential hepatotoxicity. These findings are in line with established literature indicating that VPA can adversely affect liver function, particularly in younger children or those with underlying metabolic disorders [20]. In contrast, LEV demonstrated a more favorable hepatic profile, with minimal impact on liver enzymes, but was associated with behavioral side effects, including aggression (9.7%) and irritability (8.6%). These behavioral adverse effects have been widely reported, with Taj et al. documenting that up to 12% of children treated with LEV experienced new-onset emotional lability, agitation, or insomnia [21]. Brivaracetam, a synthetic analogue of LEV, was developed to reduce the behavioral side effects of LEV but has found more roles in the treatment of focal epilepsy in both adults and children [22]. Despite these adverse effects, it is worth noting that no patient in either group required discontinuation of therapy, indicating that both drugs are clinically tolerable when carefully monitored.

These findings must be interpreted in the context of existing literature. A systematic review by Zhang et al. suggests comparable efficacy of LEV and VPA for generalized epilepsy in pediatric populations [23]. However, the review also highlighted LEV’s emerging role as a safer and more tolerable alternative, especially in patients who cannot tolerate VPA or have contraindications such as hepatic dysfunction, obesity, or metabolic disorders. In specific subtypes such as focal epilepsies, some studies suggest that LEV may even outperform VPA in terms of side effect profile and long-term adherence [22,23]. This shift reflects the broader clinical trend toward individualized treatment, where therapeutic decisions are informed not just by efficacy, but also by safety, tolerability, and patient/family preferences.

Taken together, our study suggests that VPA remains the more efficacious agent for seizure control in generalized pediatric epilepsy. Its faster onset, longer seizure-free periods, and lower dose requirements support its continued use as a first-line therapy. However, its use must be balanced against the risk of hepatotoxicity, requiring routine liver function monitoring. LEV, with its safer metabolic and hepatic profile, is a strong alternative for children where VPA is contraindicated or not well tolerated. The behavioral side effects associated with LEV underscore the need for regular neurobehavioral assessment and parental counseling, particularly during early treatment phases. Crucially, we quantify the efficacy-tolerability trade-off essential for individualized treatment. This direct, pediatric-specific comparison, along with resource utilization data like hospital stay duration, strengthens the study's contribution to clinical decision-making.

The study benefited from having comparable patient groups, a precise treatment objective, and the inclusion of EEG for validation of the seizure control. Nonetheless, its impact is constrained by several factors, including a brief follow-up of 6 months, a limited number of participants, and being conducted at a single center, which reduces statistical robustness and the ability to generalize the findings. We also did not stratify epilepsy by specific electroclinical syndromes. In the broader context, there could be differences in specific local patterns of epilepsy prevalence, patient healthcare-seeking behavior, subjective interpretation of side-effects, and drug access. It is advised that future research should involve broader, multicenter pediatric studies with extended follow-up periods and more systematic assessment of side effects and treatment adherence to more robustly validate these results.

## Conclusions

This study concludes that both VPA and LEV are effective and well-tolerated options for managing generalized epilepsy in children. However, VPA demonstrated superior efficacy in terms of longer seizure-free duration, earlier seizure control, and reduced hospital stay, despite a higher risk of metabolic and hepatic side effects. LEV, while slightly less effective, offered a better safety profile with fewer systemic adverse effects, though behavioral issues were more common. Therefore, the choice of antiepileptic drug should be individualized based on the clinical context, side effect tolerability, and patient-specific factors, with close monitoring to optimize outcomes.
